# Training need assessment for a master training program in Environmental Health program in Vietnam

**DOI:** 10.3934/publichealth.2020017

**Published:** 2020-03-23

**Authors:** Le Thi Thanh Huong, Tran Thi Tuyet Hanh, Luu Quoc Toan, Do Thi Hanh Trang, Nguyen Thuy Quynh, Nguyen Quynh Anh, Tran Khanh Long, Stanley Fenwick, Nguyen Thanh Ha, Bruce H Alexander

**Affiliations:** 1 Hanoi University of Public Health, 1A Duc Thang Rd, Duc Thang Ward, North Tu Liem District, Hanoi, Vietnam; 2 Queensland University of Technology, Brisbane, QLD, Australia; 3 Cummings School of Veterinary Medicine, Tufts University, 200 Westoboro Rd., North Grafton, MA 01536, USA; 4 School of Public Health, University of Minnesota, Minneapolis, MN 55455, USA

**Keywords:** training needs assessment, environmental health, Masters degree, Vietnam

## Abstract

Vietnam is facing a shortage of skilled Environmental health workforce. A Training Needs Assessment was conducted to develop a list of environmental health tasks, a list of core competencies and assess the need for a Master of Environmental Health training program in Vietnam. To answer these questions, a cross-sectional study was conducted in Vietnam in 2017, using both qualitative and quantitative methods. The qualitative study involved a desk review, 29 in-depth interviews, two consultative workshops, and two expert meetings. For the quantitative component, 298 environmental health staff working at different levels completed a structured postal questionnaire. Results showed that different sectors were implementing various environmental health tasks but that there was currently no training program focusing on environmental health in Vietnam. Thirteen core competencies for a Master of Environmental Health were recommended. An urgent need to develop training programs to help building environmental health competencies at the Masters degree level was uniformly expressed. This could be achieved by developing a Master of Public Health with an Environmental Health stream in the short-term and a Master of Environmental Health program in the long-term.

## 1. Introduction

Vietnam is facing an increasing number of environmental health risks due to the effects of urbanization, industrial development and rapid economic growth in the recent decades, in addition to the consequences from the use of approximately 80 million liters of defoliants used by the U.S. army during the Vietnam Wars [1]–[3]. Environmental pollution has intensified in Vietnam resulting in major concerns for human health and a large burden of diseases [3],[4]. According to the World Health Organization, environmental health addresses all the physical, chemical, and biological factors, and related factors impacting behaviors. This includes the assessment and control of those environmental factors that can potentially affect health. It is targeted towards preventing disease and creating health supportive environment [5].

After several years of development by both the Ministry of Natural Resources and Environment and the Ministry of Health, the National Environmental Health Action Plan was submitted to the Government in 2011, but was rejected in early 2012 [6]. Environmental health concerns have been raised however there is a lack of systematic and comprehensive policies pertaining to environmental health. Consequently, Vietnam is facing various environmental health problems without a comprehensive and systematic plan [6]. Several ministries, including Ministry of Health, Ministry of Natural Resources and Environment, Ministry of Agriculture and Rural Development, Ministry of Construction, and their departments at provincial and commune level, have been implementing environmental health related activities as defined by their roles and responsibilities with the purposes of protecting the health of human and the environment. However, these activities are being implemented vertically and there is a lack of coordination across ministries and sectors. Importantly, there has been weak collaboration between the health sector and the environment sector, which has resulted in reduced effectiveness of many programs and plans [1]. In general, the recognition, control, and management of environmental hazards that impact human health in the country face many challenges and are not integrated into socio-economic development policies. Additionally, poor socio-economic conditions and limited resources also inhibit solutions for handling emerging environmental health threats [1].

Environmental health science is by nature multi-disciplinary, and training programs at the Master of Science level in environmental health usually adopt a multi-disciplinary approach. Environmental health training programs are offered worldwide, however, the learning outcomes and courses emphasized in a particular program must meet the specific needs of the country in which the programs are offered. Thus, it is important to develop an appropriate Vietnamese training program in environmental health to ensure that future environmental health professionals have the knowledge, skills and competencies required to improve the health of communities in Vietnam. Although universities in Vietnam offer environmental sciences, environmental technology, and environmental management training programs, the country is currently facing a shortage of skilled human resources with environmental health expertise as training in this field is still at an early stage [1]. Environmental health training is often integrated as part of the training for other professions in public health, such as community health. While it is important for all public health professionals to have some understanding of environmental health, there is a lack of specialized training of individuals with sufficient environmental health skills, knowledge and proficiency to address the increasing environmental health challenges facing Vietnam.

Training Needs Assessment (TNA) are recommended as a fundamental process to determine the essential needs to help individuals and the organization achieve their goals and objectives [7]–[9]. In 1999, the US Health Resources and Services Administration (HRSA) established the Public Health Training Center to assess workforce-training needs and to provide skills-based training. This model was also implemented by other non-governmental public health organizations in the United States, such as the Association of State and Territorial Health Officials (ASTHO), in a similar effort to assess gaps in health competencies and to identify public health training needs [8]. In Swaziland, a training needs assessment for an M.Sc. training program in Environmental Health was carried out as part of the strategic plan for the development of postgraduate programs [7].

In 2008, the Hanoi University of Public Health (HUPH, formerly known as Hanoi School of Public Health), a leading public health training and research institution in Vietnam conducted a training needs assessment for a Bachelor of Public Health program majoring in environmental health. Since 2008, HUPH has provided this training course to meet the training needs [10]. As part of the HUPH's strategic plan for the development of a Masters program in Environmental Health to address the needs of Vietnam, a training needs assessment was carried out in 2017 to (1) develop a list of environmental health tasks that require staff with a Masters degree, (2) develop a list of environmental health core competencies for environmental health staff with a Masters degree and (3) assess the training needs at the master level for a Master of Environmental Health in Vietnam.

## 2. Materials and methods

The study was conducted in 2017 in seven cities/provinces, which were representative of seven socio-economic regions of Vietnam, including Dien Bien, Hanoi, Ha Tinh, Binh Dinh, Dak Lak, Hochiminh city and Can Tho. This cross-sectional study used a mixed-methods approach with qualitative and quantitative components. The qualitative survey included 29 in-depth interviews and two focus group discussions (8–10 participants per discussion). In addition, there were two consultation meetings using Delphi technique with 20 experts from universities, managers and staff national level, and 17 experts from health, environment, agriculture and rural development sectors at provincial level. The quantitative component of the study included sending self-administered questionnaires to 361 employees who were working in the fields related to Environmental health at national and provincial levels. 298 completed questionnaires were returned, resulting in a response rate of 82.5%.

The first and second study objective were addressed using the qualitative approaches. The first study objective was to identify environmental health activities at the national and local levels, the environmental health activities that need expertise at the masters level, and identifying the competencies required for the environmental health workforce. The second objective was to propose a list of environmental health core competencies that staff working in the environmental health field were satisfied at master level. The competencies include assessment competency, management competency, communication competency. The focus groups and workshops discussed the suitability, feasibility and the gaps of the Environmental health core competencies.

For the third objective, a quantitative questionnaire was developed based on the competencies identified in the second objective. The questionnaire was developed to ascertain the training needs for the environmental health core competencies upon which a master of public health tracking in environmental health training program could be developed. Each competency was subjectively assessed by the participants using three scales: “no need training”, “need training”, and “urgently need training”. The quantitative data was managed by EpiData 3.1 software and was analyzed by Stata 14.0 software. The qualitative data was analyzed according to themes. The study received approval from the Ethical Review Board of Hanoi University of Public Health (No. 297/2016/YTCC-HD3).

## 3. Results

### 3.1. Study population

220 participants (73.8%) had training at bachelor level and the remainder were at master level. Nearly a third (29,9%) worked at national level and the remaining participants worked at provincial level. Approximately two-thirds (67.4%) of the participants worked for the health sector and 32.6% worked for the environment sector. The distribution of work experience of the participants was 1 year and less (37.3%), 2 to 5 years (28.2%), 6 to 10 years (22.1%) and more than 10 years (12.4%).

### 3.2. Environmental health tasks in Vietnam that require staff at master level

The assessment showed that the list of the environmental health tasks to be conducted at different levels in the health system in Vietnam that required staff at master level could be classified into three major task groups related to: (1) assessment, mornitoring and research, (2) management, planning and implementation, and (3) communication, training and reporting (Table 1).

**Table 1.** Environmental health tasks in Vietnam requiring staff trained at the master's level.

**STT**	**Environmental health tasks in Vietnam requiring staff at master level**
**I.**	***Tasks related to assessment, mornitoring and research***
1	Monitoring air, water, soil quality, medical wastes and human health impacts.
2	Identifying and providing warning on environmental health hazards which pose health risks to the communities.
3	Developing, piloting and implementing tools for assessing and forecasting environmental pollution risks.
4	Conducting health impact assessments, environmental health risk assessment, and environmental impact assessment.
5	Assessing impacts of climate change on human health and socio-economic development; Assessing impacts of global climate change policies on socio-economic development in Vietnam; Participating in greenhouse gases inventory and environmental auditing.
6	Investigating, and assessing scope and levels of health impacts due to environmental issues at hot spots and propose intervention measures.
**II.**	***Tasks related to management, planning and implementation of programs/activities***
1	Assessing, providing early warning of and managing environmental incidences.
2	Managing general wastes and medical wastes.
3	Conducting scientific research related to health impacts of air/water/soil pollution, climate change, medical wastes, etc.
4	Planning and implementing drinking water quality monitoring and supervising the implementation of water and sanitation programs/activities.
5	Collaborating with Department of Environmental Protection to implement environmental monitoring activities
6	Collaborating with environmental policies to implement environmental and hygiene inspection at premises.
7	Collaborating with local authorities and related sectors in responding to environmental incidences.
8	Developing documents and policies related to environmental health
**III.**	***Tasks related to communication, training and report***
1	Communicating on environmental health issues, programs, projects (e.g. washing hands with soap, hygienic toilets, risk communication, communication on environmental protection…)
2	Training on medical waste management, water and sanitation, school health etc.
3	Participating in the preparation of national environmental health report
4	Developing and updating the national environmental health profile

Results presented in Table 1 show that although there were currently no official training programs for bachelor and master of environmental health, different sectors in Vietnam were currently implementing various environmental health tasks. In depth interviews results showed that the participants felt that a need existed for master of science (MSc.) degree qualified personnel in the field of environmental health in their respective organizations to ensure the quality of these task implementations, especially those with the position “Head of Environmental and Community Health Department” at the Provincial Department of Preventive Medicine/provincial Center for Disease Control and Prevention (CDC). Some organizations have specialized areas such as medical waste management, drinking water quality monitoring, etc. that need the service of environmental health specialists, but currently there was no one with a master's level training in environmental health to fulfill planning and decision-making tasks. Several organizations anticipated the need for masters level training with a focus on environmental health in order to carry out some specialized environmental health tasks, such as environmental health risk assessment, risk communication, climate change adaptations, which were not trained in other existing training programs. The competencies for Bachelor of Public Health are not sufficient to cover all these tasks. In Vietnam, the competencies for Master of Public Health or Bachelor of Public Health majoring in Environmental Health are not yet officially developed and approved.

### 3.3. Environmental health core competencies for staff at master level and the situation of training for these competencies

Based on a review of competencies for environmental health professionals in other countries, current Vietnam Government's documents on tasks and responsibilities of staff working in environmental health related fields and results of in-depth interviews, a list of potential core competencies for environmental health staff at a master' level was developed. Corresponding to the required tasks presented in Table 1, the potential core competencies were categorized in four main groups including “assessment and monitoring competencies”, “management competencies”, “communication competencies”, and “others”. Assessment of the appropriateness of these competencies for staff at national and provincial levels was discussed among environmental health experts in the consultation meetings and the results are presented in Table 2 below.

**Table 2.** Assessment of the appropriateness of potential core Environmental health competencies as reported by focus group discussions and expert meetings.

**Potential competencies**	**Appropriateness**	
	***National level***	***Provincial level***
**Assessment and monitoring**		
1. Conducting complicated tests for water and air quality assessment	+	+
2. Identifying and quantifying environmental toxicants	++	+
3. Conducting health impact assessment and environmental impact assessment for programs/projects	+++	++
4. Assessing environmental health risks	+++	+++
5. Assessing vulnerability and adaptability to climate change of different population groups.	+++	+++
**Management**		
1. Managing environmental health risks	+++	++
2. Managing solid wastes and hazardous wastes	+++	+++
3. Monitoring and controlling water and air pollution	+++	+++
4. Responding to environmental health emergencies	++	++
5. Applying GIS and remote sensing techniques in managing and monitoring environmental health problems including environment-related diseases and emerging infectious diseases.	++	+
6. Providing early forecast and warning of health and environmental impacts of climate change and recommending solutions to these impacts.	+++	++
**Communication**		
1. Communicating about environmental health risks with the community and related authorities timely, honestly and objectively	+++	+++
2. Providing effective consultation on environmental health interventions, minimizing environmental impacts on health based on environmental health data.	+++	+++
3. Resolving environmental health conflicts effectively	+++	+++
4. Conducting environmental health marketing.	+++	+++
**Others**		
1. Having good knowledge about laws, environment and epidemiology	++	++
2. Possessing soft skills (e.g. teamwork skills, communication skills, language skills, computer skill, etc.)	+++	+++

Note: +++ very appropriate, ++ appropriate, + a little appropriate

According to the environmental health experts involved in the discussion, almost all of these potential competencies were important for masters in environmental health graduates working at both national and provincial levels, and important to both managers and staffs. However, the level of application of each competency varied depending on their working positions. The participants agreed that competencies which involved highly technical skills such as “Applying GIS and remote sensing techniques in managing and monitoring Environmental health problems including environment-related diseases and emerging infectious diseases” were applicable to those working at national level but little applicable at provincial level.

Based on the results of the literature review, in-depth interviews with environmental health personnel (both managers and staff) and consultation with environmental health experts, the list of core competency recommended for a master of environmental health was finalized and presented in Table 3 below. This list involves 13 competencies, clustered into three groups namely “environmental health risk assessment”, “environmental health management”, and “risk communication, work-related communication and education”.

**Table 3.** Final list of core competencies for master of Environmental health recommended for Vietnam.

**No**	**Core competencies for master of Environmental health**
**I**	***Environmental health risk assessment***
1	Assessing Environmental health risks
1.1	Planning and implementing risk assessment related to chemicals (e.g. chemicals present in water, soil, air, and food)
1.2	Planning and implementing quantification assessment of microbiological risks (e.g. those in water or food)
2	Assessing health impact and environmental impact of programs/projects/policies
2.1	Planning and implementing environmental impact assessment following existing legal documents
2.2	Planning and implementing health impact assessment complying with existing legal documents
3	Identifying and assessing health risks of climate change and extreme weather events
3.1	Identifying determinants of climate change
3.2	Assessing impacts of climate change and extreme weather events on human health
3.3	Assessing vulnerability and adaptation capacity related to climate change and extreme weather events
**II**	***Environmental health management***
4	Providing consultation on solutions to environmental health emergencies
5	Managing Environmental health risks
6	Effectively collaborating with partners and related sectors in doing research and managing environmental health issues
7	Planning and implementing water quality monitoring, waste management, medical waste management, and injury intervention in community settings
8	Applying information technology (e.g. GIS) in managing and monitoring environmental health issues, environment-related diseases and emerging infectious diseases.
9	Collecting and analyzing information and writing reports related environmental health
**III**	***Risk communication, communication at workplace and education***
10	Training staff from lower levels in environmental health related issues
11	Communicating health Environmental health risks to the community and other stakeholders timely, honestly, effectively and objectively.
12	Effectively applying soft skills (communication, decision making, team work, etc.) in work and resolving conflicts related to Environmental health
13	Synthesizing and communicating effectively research findings, program or project reports to stakeholders for use in making decisions and in developing, implementing and evaluating policies related to Environmental health.

### 3.4. The training needs for 13 environmental health core competencies

**Figure 1.**
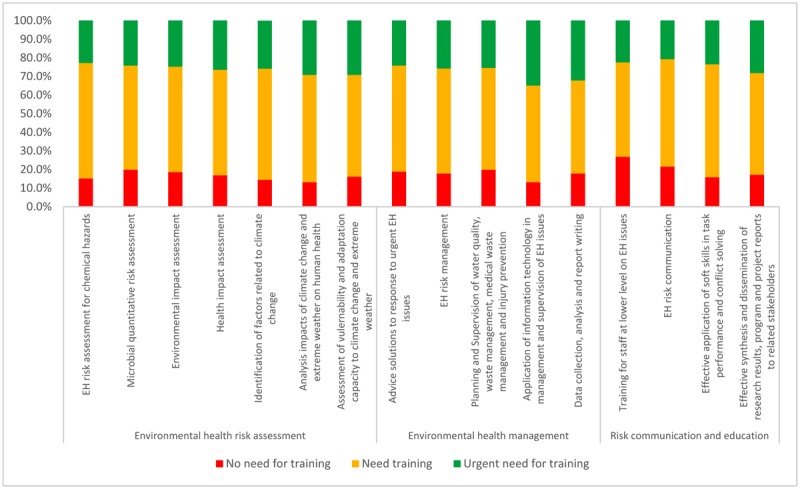
Training need assessment for Environmental health core competencies in Vietnam.

Results regarding training needs for the 13 environmental health core competencies (in which competencies 1, 2, 3 were divided into sub-competencies) are presented in Figure 1. The training needs were relatively high with levels from “need training” to “urgent need for training” for each competency being reported by approximately 80% of the participants or more. The level of “urgent need for training” for every competency was reported by more than 20% of the participants. In general, there were no significant differences in the prevalence of “need training” and “urgent need for training” for Environmental health core competencies between staff at national and provincial level, except for the competency “Planning and supervision of water quality, waste management, medical waste management and injury prevention”, with the higher prevalence of “need training” and “urgent need for training” belonging to provincial staff.

The expected training modes included short courses and an official Master of Environemntal Health. In total, there were 40.9% of staff expected to undertake the master program if the program would be developed and delivered by HUPH, and the remaining of the participants hoped to attend short courses that covered the environmental health topics they wanted to be trained. Participants were also asked to provide a list of five most important competencies that should be prioritized for training in the future master of environmental health at HUPH. Table 4 presents the prevalence of participants at national and provincial levels reporting each prioritized competency. There was much similarity between the list reported by staff at national level and that by staff at provincial level. Only the competency “*Analysis impacts of climate change and extreme weather on human health*” was not reported by national staff, but appeared in the list of provincial staff, and the competency “*Environmental health risk management*” can only be found among the list of five most competencies of the national staff. “*Effective application of soft skills (communication, decision making, teamwork, etc.) in task performance and Environmental health conflict solving*” seemed to be the most important competency for staff at provincial level. The competency “*Data collection, analysis and report writing in Environmental health*” also seemed to be the most important one for national staff.

**Table 4.** The five most important competencies that be prioritized for training at the national and provincial level.

**TT**	**The five most important competencies**	**N**	**%**
	**All staff (n = 213)**		
1	Effective application of soft skills (communication, decision making, teamwork, etc.) in task performance and environmental health conflict solving	97	45.5
2	Data collection, analysis and report writing in environmental health	83	39.0
3	Environmental health risk assessment	80	37.6
4	Effective synthesis and dissemination of research results, program and project reports to related stakeholders	76	35.7
5	Analysis impacts of climate change and extreme weather on human health	62	29.1
	**Staff at national level (n = 59)**		
1	Data collection, analysis and report writing in environmental health	27	45.8
2	Effective application of soft skills (communication, decision making, team work, etc.) in task performance and environmental health conflict solving	25	42.4
3	Effective synthesis and dissemination of research results, program and project reports to related stakeholders	24	40.7
4	Environmental health risk assessment	22	37.3
5	Environmental health risk management	22	37.3
	**Staff at provincial level (n = 154)**		
1	Effective application of soft skills (communication, decision making, teamwork, etc.) in task performance and Environmental health conflict solving	72	46.8
2	Environmental health risk assessment	58	37.7
3	Data collection, analysis and report writing in Environmental health	56	36.4
4	Effective synthesis and dissemination of research results, program and project reports to related stakeholders	52	33.8
5	Analysis impacts of climate change and extreme weather on human health	44	28.6

#### 3.4.1. Important competencies should be covered in future environmental health program

Results from the qualitative data collection were consistent with what were observed in the quantitative survey, with similar competencies needed to be trained mentioned by the study participants. Based on the Delphi technique results with experts from universities, managers and experts at national and provincial levels, it revealed that all the important competencies should be covered in the future master program of environmental health at HUPH.

“*The teaching content should cover the causes of environmental health risks, the measures to prevent and mitigate those risks. Especially, the program should focus on planning and management of the risks, as well as other important tasks such as water quality surveillance, monitoring of latrines, and training for staff at lower levels*” (Male participant, working at provincial level).

“*I think the component of environmental health risk assessment, management and communication should be the most important. Health impact assessment and environmental impact assessment are also important too*” (Female participant, working at provincial level).

Concurrent needs for the master program of EH and master program of public health majoring in EH

Results from in-depth interviews showed that there existed training need for master programs related to environmental health. For staff working in the health sector, the need for the master of public health majoring in environmental health was more urgent than the need for the master of environmental health since in Vietnam, currently there were no official environmental health professional. However, in the environmental sector, there was a need for the master of environmental health. The need for the master of public health program majoring in environmental health was an urgent need in the short term, while the need for the master of environmental health should be a focus for the HUPH in the next few years, because it would take a few years of preparation for HUPH to get approval from the Ministry of Education and Training (MOET) for this program.

“*I think both the programs [Master of Public Health program majoring in Environmental Health and the Master of Environmental Health program] are important, and I propose that the university [HUPH] should immediately develop the master program of environmental health, because graduates of this program can response to various environmental health tasks at different sectors such as health, environment, and agricultural and rural development sectors, and they can fulfill the gaps of insufficient qualified staff in environmental health currently*” (Male participants/policy makers, working at central level).

## 4. Discussion

### 4.1. List of environmental health tasks currently implemented in Vietnam, challenges and opportunities

On a global scale, countries are facing with the challenges of addressing the burden of diseases arising from environmental exposures. Capacity building in environmental health has been recognized as a critical need in different countries [11]. The results of this TNA assessment showed that there was a strong need for trained professionals to implement various tasks of environmental health to deal with existing and emerging environmental health problems in Vietnam. However, since there was currently no official position called “Environmental Health Officer” in the governmental system in Vietnam, the demand for environmental health professionals remains quite low. There were, however, a list of various tasks on environmental health, which were currently implemented by health staff and staff working in related sectors, including Natural Resources and Environment, Agriculture and Rural Development, Construction, etc. Similar situations were observed in other countries, where low demand for environmental health professionals leads to low intake rates for training in universities while the reality of environmental health problems indicate that there was a high need for trained professionals [12]. This was one of the current challenges since the heads of departments and staff dealing with different environmental health activities did not have a master or bachelor degrees in environmental health. Senior officers and/or managers of Department of Environmental and Community Health at Provincial CDC in Vietnam have to implement various environmental health tasks as described in Table 1, but without the necessary environmental health training and qualifications. Although they may have a master degree (e.g. Master of Public Health), they lack technical skills concerning environmental issues and thus face challenges in ensuring the quality of implementing various environmental health tasks. The list of tasks described in Table 1 is important in informing the development of competencies and learning outcomes for training programs that meet the specific needs in Vietnam. This TNA results informed the development of environmental health training programs that incorporated priority environmental health problems and meet the specific need of implementing environmental health tasks in Vietnam.

### 4.2. Environmental health core competencies for staff at master level and the situation of training for these competencies

The development of the list of core competencies for masters of environmental health was undertaken through several stages, with careful consideration of internationally recommended competencies for environmental health human resources at this level and also the appropriateness of these competencies to the Vietnamese context. Similarly to the core competencies for Environmental health professionals recommended by developed countries including the US [13] and Canada [14], the competencies recommended for Vietnamese environmental health professionals with a Master's in Environmental Health degree also included three areas; risk assessment, management and communication. However, the two mentioned international lists of core competencies do not distinguish environmental health human resources based on levels of qualification. In Vietnam, there are no training programs or any governmental documents that provide a list of core competencies required for a Bachelor of Environmental health graduate. However, HUPH, as a leading institution in public health training and research in Vietnam, is providing a Bachelor of Public Health (BPH) training program with a major on environmental health that specified environmental health related competencies for the graduates from this program [10]. Compared to a BPH majored in Environmental Health, Master of Environmental Health graduates are expected to possess more environmental health specialization and more advanced skills and knowledge; e.g. those related to health impact assessment and environmental impact assessment, vulnerability assessment, risk communication, Environmental health risk assessment, application of GIS and remote sensing technology in management of environmental health issues including emerging infectious diseases, early forecasting and warning of environmental health impacts of climate change, resolution to environmental health conflicts and environmental health marketing. These competencies are different from those included in the Professional Competencies of Bachelor of Public Health in Vietnam, which was recently approved and also different from the learning outcomes of master of Public Health training programs offered in Vietnam.

### 4.3. Training needs for environmental health competencies at different levels

The results found in this study showed a strong training need for Environmental health competencies. These findings were similar to what were found in the report of “Training need assessment for the Bachelor program of public health majoring in environmental and occupational health”, with low proportion of staff to be trained officially in environmental health competencies [10]. The results also revealed a fact that there was no official program in Environmental health at both undergraduate and postgraduate levels in Vietnam, except for the bachelor of public health program majoring in environmental and occupational health at HUPH [1]. These results also implied that there was a lack of qualified environmental health staff in Vietnam, which was similar to other low- and middle-income countries such as Swaziland [2],[7].

The training need assessment results also showed the needs for both the short training courses and the official master program training (both Master of Public Health program majoring in Environmental Health and Master of Environmental Health program) to meet the current and future needs of various staff working in environmental health in Vietnam. The Master of Public Health majoring in environmental health would satisfy the training needs for health staff working in environmental health areas, while the Master of Environmental Health requiring time for approval from MOET would meet the need for staff working in the environment sector, and these two programs would provide qualified environmental health staff to fulfil the gap which had been indicated by Le Thi Thanh Huong et al. in 2015 [1]. The development of these training programs with the above-mentioned environmental health competencies is also appropriate with the recent emphasis on the application of a multidisciplinary One Health approach in addressing complex environmental health challenges in Vietnam, especially those that occur at the intersection of humans, animals and the environment, such as food safety and security, water quality, and emerging and re-emerging zoonotic diseases.

Based on the study results, a MPH program majoring in Environmental Health was developed at the Hanoi University of Public Health and was approved by the University's Education and Scientific Committee, with 15 credits majoring in EH. The program began recruiting students for the academic year 2019-2020. Core competencies for the MPH majoring in EH identified by this study were integrated into the program, such as “environmental health risk assessment”, “environmental health risk management”, “Risk communication, communication at workplace and education” as shown in Table 3.

### 4.4. Study's limitations

This was the first study in Vietnam on training need assessment for master of environmental health in Vietnam and provided important information to inform the training program development. However, several limitations should be taken into consideration when interpreting the findings of this study. Since this was the first study of its kind implemented in Vietnam, there was no validated tool for the assessment of training needs available. Similar studies implemented in other countries were relevant, but not fully applicable. In addition, there was currently no formal environmental health position in Vietnam and this was a field that involved different ministries/sectors, such as Natural Resource and Environment, Health, Agriculture and Rural Development etc. Thus, the study might not have explored perspectives of all related sectors in assessing the training need nor could provide estimated numbers of staff who wished to undertake Master of Environmental Health or Master of Public Health, majoring in Environmental Health in the next 5–10 years and in the longer term. Nevertheless, to ensure a comprehensive assessment, the study included a wide range of participants and applied both quantitative and qualitative research methods. Another limitation of this study is that 73.1% of the participants who completed the questionnaire came from the Health Sector and only 26.9% working in the Environmental Sector. Thus, the quantitative results may not truly reflect the actual training need in the country. However, the research team tried to overcome this limitation and to have a more comprehensive picture of the training need by conducting in-depth interviews with participants coming from different sectors, including Health, Environment, Environmental Police, Agriculture and Rural Development etc.

## 5. Conclusion and recommendations

Quantitative and qualitative results of this training need assessment showed that there was an urgent need in developing training programs to help building competencies in environmental health at master level for staff working in the health, environment, and related sectors in Vietnam. To meet the current urgent need, HUPH and other universities should consider developing master programs related to environmental health, which should be conducted in two periods. For the next five years, the Master of Public Health majoring in environmental health, which is based on the current master of public health program, should be developed and implemented. In the longer term (e.g. in the next 5 to 10 years), the Master of Environmental Health program should be developed to meet the training needs in the country. The new master of Environmental health program needs to assure that graduates will acquire 13 Environmental health core competencies at master level, which belong to three main groups identified in this assessment.
